# Electrically focus-tuneable ultrathin lens for high-resolution square subpixels

**DOI:** 10.1038/s41377-020-0329-5

**Published:** 2020-06-05

**Authors:** Sehong Park, Gilho Lee, Byeongho Park, Youngho Seo, Chae bin Park, Young Tea Chun, Chulmin Joo, Junsuk Rho, Jong Min Kim, James Hone, Seong Chan Jun

**Affiliations:** 1 School of Mechanical Engineering, Yonsei University, 50 Yonsei-ro, Seodaemun-gu, Seoul, 03722 Republic of Korea; 2 Electrical Engineering Division, Engineering Department, University of Cambridge, 9 JJ Thomson Avenue, Cambridge, CB3 OFA UK; 3 Department of Electronic Material Engineering, Korea Maritime and Ocean University, Busan, 49112 Republic of Korea; 4 Department of Mechanical Engineering, Pohang University of Science and Technology (POSTECH), 77 Cheongam-ro, Nam-gu, Pohang, 37673 Republic of Korea; 5 Department of Chemical Engineering, Pohang University of Science and Technology (POSTECH), 77 Cheongam-ro, Nam-gu, Pohang, 37673 Republic of Korea; 6 Institute for Convergence Research and Education in Advanced Technology, Yonsei University, 50 Yonsei-ro, Seodaemun-gu, Seoul, 03722 Republic of Korea; 7 Department of Mechanical Engineering, Columbia University, 500 West 120th Street, Mudd 220, New York, NY 10027 USA

**Keywords:** Optoelectronic devices and components, Displays, Optical properties and devices, Electronic properties and devices

## Abstract

Owing to the tremendous demands for high-resolution pixel-scale thin lenses in displays, we developed a graphene-based ultrathin square subpixel lens (USSL) capable of electrically tuneable focusing (ETF) with a performance competitive with that of a typical mechanical refractive lens. The fringe field due to a voltage bias in the graphene proves that our ETF-USSL can focus light onto a single point regardless of the wavelength of the visible light—by controlling the carriers at the Dirac point using radially patterned graphene layers, the focal length of the planar structure can be adjusted without changing the curvature or position of the lens. A high focusing efficiency of over 60% at a visible wavelength of 405 nm was achieved with a lens thickness of <13 nm, and a change of 19.42% in the focal length with a 9% increase in transmission was exhibited under a driving voltage. This design is first presented as an ETF-USSL that can be controlled in pixel units of flat panel displays for visible light. It can be easily applied as an add-on to high resolution, slim displays and provides a new direction for the application of multifunctional autostereoscopic displays.

## Graphene: ultrathin tuneable lens

Graphene-based ultrathin lenses with an electrically tuneable focal length could prove useful for providing displays with autostereoscopic 3D functionality, multiview or privacy protection. Developed by scientists in South Korea, the UK and the US, the lenses are just 13 nm thick and are based on a Fresnel Zone Plate (FZP) design made from a series of concentric rings of graphene on a glass substrate. Consisting of up to 5 layers of graphene that is patterned by focused ion-beam milling, the lenses offer a 60% transmission in the visible region and a focal length of 200–300 µm that can be tuned within ~20% range by applying a DC bias voltage. The applied voltage changes the charge carrier density in the graphene, modifying the topology of the FZP and thus its focusing behaviour.

## Introduction

Traditional tuneable lenses^1^ consisting of complex lenses with manipulation systems have limited designs because of the spatial occupancy, which eventually confines their applications in advanced pixel-based devices, such as flat panel displays. Ultrathin flat lenses, which are different from conventional lenses, have been extensively investigated owing to the growing interest in ultrasmall devices, with various applications in telescopes^2^, aerospace technology^3^, holograms^4^, and displays^5^. Realisation of ultrathin flat lenses has been achieved using diffractive optical elements^6,7^ or metasurfaces^8,9^. In particular, Fresnel zone plates^10^ (FZPs) comprise a planar ring pattern and can be designed as thin, low-volume, and high-numerical-aperture lenses for applications in various fields, such as ultrathin nanolithography^11–13^, near/far-field optical microscopy^14,15^, optical antennas, and X-ray optics^16–19^. For highly efficient Fresnel lenses, a 200-nm thick graphene-oxide-based FZP with an absolute focusing efficiency of 32% has been developed^7^. In the case of ultrathin films, an FZP with a radius of 40 µm and 24 zones employing multilayer graphene was reported to have a thickness of 3.47 nm for ten layers, and a focusing efficiency of 6.6% with transmittance in the range of 75–78% (ref. ^6^). Additionally, an FZP with nanoribbons can achieve high intensity and efficiency owing to the optical plasmon coupling phenomenon, depending on the width of the nanoribbons and the carrier concentration of the graphene^20,21^. Hence, a material that significantly enhances the current ultrathin lenses through higher transmittance, higher focusing efficiency. and lower thickness is essential.

Graphene and graphene composites have dynamic optical properties and unique electrical properties. Because the interlayer interaction is negligible for monolayer graphene, it is transparent in the visible light regime. Meanwhile, few-layer graphene with <10 layers exhibits light absorption characteristics linearly proportional to the number of graphene layers *N*, as the absorption *A*(*ω*) = 1−*T*(*ω*) = *παN*, with fine-structure constant *α* (refs. ^22,23^). This property facilitates control of visible light transmittance by varying the number of graphene layers. In addition, the transmittance of graphene is determined by the optical conductivity (i.e., dynamic conductivity) *G*(*ω*), which is correlated with the Fermi energy *E*_*F*_ and the carrier density *n* induced by an external electric field, as the integrated absorption ${\int} {\left| {\Delta G_{{\mathop{\rm{int}}} ra}} \right|} d\omega ,{\int} {\left| {\Delta G_{{\mathop{\rm{int}}} er}} \right|} d\omega \propto \sqrt {E_F/m_{e,h}^ \ast } \sqrt n$ (ref. ^24^). These properties represent potential tuneability in the optical response regime^25^. In fact, owing to the tuneable characteristics of graphene, graphene-based devices, such as optical waveguides^26^, polarisers^27,28^, optical modulators^29^, and photodetectors^30^, have been developed because the charge density of graphene can be easily varied by chemical doping, gate biasing^25^, and changing the magnitude and direction of an externally applied electric field^31^. Therefore, if the graphene can be patterned into nanoribbons^32^, then a graphene-based FZP lens can be an ideal combination of near and far optical fields because the optical conductivity of graphene can be tuned by adjusting the Fermi level or by varying the geometry. In the past, the lenticular lens^33^ and parallax barrier^34^ used in multiview autostereoscopic displays were considered infeasible in displays owing to their thickness, low transmittance, high aberration, and low resolution. However, the Fresnel lens made of graphene enables electrically tuneable focusing (ETF) based on the difference in the absorption characteristics of the Fermi level; thus, a multifunctional display using an ultrathin square subpixel lens (USSL) of high transmittance and high resolution can be realised. In this study, we developed an ETF-USSL with a transmittance of over 80% for displays with a thickness of 13 nm, using the focused ion beam (FIB) method^35^ to create a nanoscale pattern on a thin graphene structure. The results indicate that the graphene can be used to change the viewing angle of the display via dynamic control of the focal length with a DC voltage bias. When an ultrathin lens with an ETF is used in a display, it is possible to achieve an autostereoscopic display with multiple functions^36^, such as glassless three-dimensional (3D) viewing, privacy protection, and multiview, depending on the variation of the focal length.

## Results

### Concept of a tuneable focal length of an ultrathin subpixel lens

**Fig. 1:**
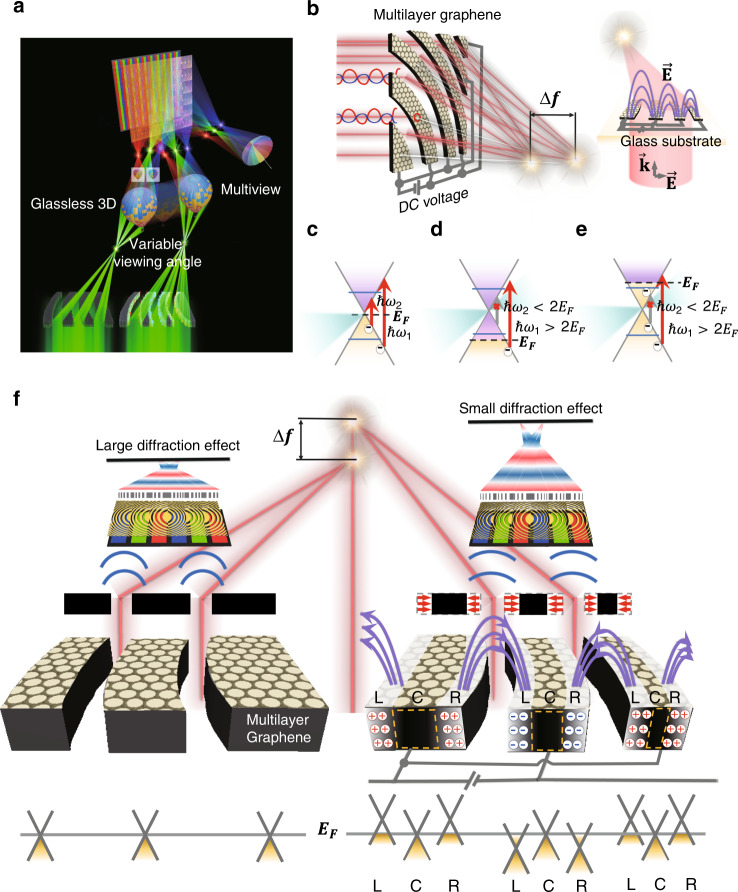
Mechanism of a graphene-based tuneable focal length. **a** Illustration of applying the ETF-USSL in a display. The USSL enables multifocusing, allowing implementation of glassless 3D and multiview displays, and the ETF characteristics enable a variable viewing angle. **b** Illustration of focusing through the graphene arc ribbon pattern. Graphene’s conical band structure and photon absorption transition with a shift of the Fermi level (*E*_*F*_) from the Dirac point due to a DC voltage bias: **c** the optical absorption of graphene increases owing to the occurrence of interband transitions, resulting from the excitation of electrons by optical photons (*ħω*_2_), where the Fermi level is close to the Dirac point, resulting in decreased transmittance. **d** The Fermi level drops to below the transition threshold; because there are no electrons available for transition, the transmittance of the graphene increases. **e** The Fermi level rises above the transition threshold; the absorption is reduced by Pauli blocking of the interband transition, and the transmittance of the graphene increases. **f** Schematic of the tuneable focal length when a DC voltage bias is applied to graphene in the in-plane direction. In the ribbon made of graphene, the centre area (C) absorbs the light, and the carrier are concentrated in the left side (L) and right side (R) due to the DC bias; thus, the Fermi level is far from the Dirac point, and light is not absorbed and transmitted. Consequently, the change in the nanoribbon width via an external electric field effectively modulates the FZP topology, thereby changing the focal length of the lens

A graphene ultrathin pixel lens was designed considering the field of view (FOV) of a multifunctional autostereoscopic display. The FZP-based USSL with tuneable focusing capability was developed to realise multifunctional lenses. The structure of the ETF-USSL was determined based on the desired optical path of a multifunctional system, based on the same frame as that of the subpixels. It is thus possible to realise glassless 3D and privacy images, when the ETF-USSL changes the optical paths of view 1 and view 2 from the near field to the far field within a single display. Therefore, the optical path of our lens was designed according to the FOVs of view 1 and view 2 for the conventional Fresnel lens by considering the subpixel size in the display panel. For two red–green–blue/red–green–blue (RGB/RGB) pixels, the RGB subpixel for view 1 on the right side (or the right eye) and the neighbouring pixel for view 2 on the left side (or the left eye) are designed such that they can be fitted into the pixel structure of a Fresnel lens pattern (more information is provided in Supplementary Fig. 1). Ultimately, the multifocusing performance of the USSL allows the implementation of glassless 3D and multiview displays, and the ETF characteristics allow the realisation of variable viewing angles for 3D images, as shown in Fig. 1a.

We therefore prepared a graphene-based USSL with an arc ribbon pattern for the application of an electromagnetic field, supported by the beneficial optical properties of graphene. The external electrical variation^37^ obtained by applying a voltage bias can control the optical properties of graphene via the change in the Fermi level. The optical conductivity^38,39^ for a thin-film model of a graphene sheet can be obtained from the Kubo model^40^ (*σ* = *σ*_intra_ + *σ*_inter_). In the Kubo model, the Fermi level depends on the change in the carrier density due to an electric field. Because the optical conductivity in the intraband is proportional to the Drude weight ${\mathrm{D}} = (v_Fe^2/\hbar )\sqrt {\pi n}$, with Fermi velocity *v*_*F*_, the carrier density *n* increases as the optical conductivity increases. However, as the carrier density related to the Fermi level increases, the optical conductivity in the interband decreases due to interband Pauli blocking^24^. To clarify the relation between the optical conductivity and carrier density, the integrated absorption in the Kubo model is ${\int} \Delta G_{intra}^\prime + \Delta G_{intra}^\prime d\omega = (v_Fe^2/2\hbar )\sqrt {\pi n} - (e^2/2\hbar ^2)E_F$, referred to as the charge neutral point. Thus, an arc ribbon made of graphene can be used to control the focal length of the lens, as the Fermi level varies when a DC voltage bias is applied to graphene in the in-plane direction, as shown in Fig. 1b. Graphene has a unique band structure with a Dirac point and electron and hole conical bands. In particular, the multilayer graphene used in our system exhibits a conical-based band structure with a small gap, which is crucial in the variation of the transparency by shifting the Fermi level using an external electrical field^25,31^. The transparency of graphene decreases when the Fermi level moves near the Dirac point because the photons from the incident light are absorbed and excite the electrons (Fig. 1c). On the other hand, when the Fermi level is located far from the Dirac point (*ħω*_2_ < 2*E*_*F*_), the transparency of graphene is increased; as shown in Fig. 1d, a high photon energy is required to excite an electron if the Fermi level is too low. It can also be found in Fig. 1e that if the Fermi level is in the conduction band (*ħω*_2_ < 2*E*_*F*_), then no absorption occurs^29^. Therefore, the electric field normal to the plane due to the DC bias concentrates the carrier density at the edges of the arc ribbon. The arc ribbon then absorbs light in the central area (C), but the Fermi level moves away from the Dirac point, owing to the increase in the carriers on the left (L) and right (S) sides, as shown in Fig. 1f. This results in a longer focal length because the decrease in the size of the arc ribbon increases the linearity of the diffraction by the arc ribbon (Fig. 1f).

### Design of a graphene-based single USSL

A single USSL was designed considering the focal length for a multifunctional autostereoscopic display (Supplementary Fig. 2). To verify the performance of the USSL, the horizontal size (*h*_*p*_) of the subpixel was set to 26 μm, and the FOV was set in the range of 2°–12°. Under these conditions, the calculated value of the focal length of a single USSL is 140 μm. This result determines the radius of the *m*th ring of the USSL. The radius is designed considering the light wavelength of a single USSL, and it must satisfy the following condition^7^ (Supplementary Note 1):

1 $$r_m = \sqrt {2\lambda f\left( {m - \frac{{\Delta \varphi }}{{2\pi }}} \right)} .m\lambda \ll f$$

The phase difference is Δ*φ* = 2Δ*nπt*/*λ* considering the factor of the initial phase shift of the harmonic wave^41^, where Δ*n* is the refractive index difference, and *t* is the thickness of a single USSL. As the equation above indicates, *r*_*m*_ depends on the incident wavelength (*λ*) and the focal length (*f*), assuming that Δ*φ* is insignificant in the calculation.

The maximum value of *r*_*m*_ can be obtained from the subpixel size of the display; thus, a single USSL was designed by matching the subpixel size (*h*_*p*_ × *v*_*p*_) to the area of the FOV for the developed symmetrical FZP ring lens (Fig. 3d). Here, *θ*_1_ and *θ*_2_ are the smallest and largest angles for the desired FOV, and *c* is the side length in forming the angle *θ*_1_ with the focus. The design variables *h*_*p*_ and *c* can be altered, while maintaining a constant focal length, but *θ*_1_ and *θ*_2_ must be accordingly changed to satisfy the mathematical relation $f = (h_p + c)/tan\theta _2 = c/tan\theta _1$, leading to a change in the USSL pattern. The obtained ring parameters of each subpixel lens are summarised in Supplementary Table 1, where the distance between the ring patterns is on the nanoscale, with a minimum value of 680 nm (between *r*_19_ and *r*_20_) for the red USSL.

Based on the Rayleigh–Sommerfeld theory^41^, we built a simulation model for our diffraction pattern to analyse the focusing performance of a single USSL. We simulated three different types of designs: a conventional ring, an arc ribbon, and a cylinder. This simulation allows the study of the paraxial intensity distributions in the focal region throughout the subpixel. According to the simulation, the focus was formed on the designed focal plane at 140 μm, where the subpixel lens of the arc ribbon type showed the same results as the conventional ring type. On the other hand, the subpixel lens of the cylindrical type showed multifocal spots in the designed focal plane (more information is provided in Supplementary Fig. 3). Consequently, these results demonstrate that our ultrathin flat pixel lens can be applied to realise an USSL with fine focusing characteristics, and with a structure fit for display pixels.

### Optical characteristics of a graphene-based single USSL

**Fig. 2:**
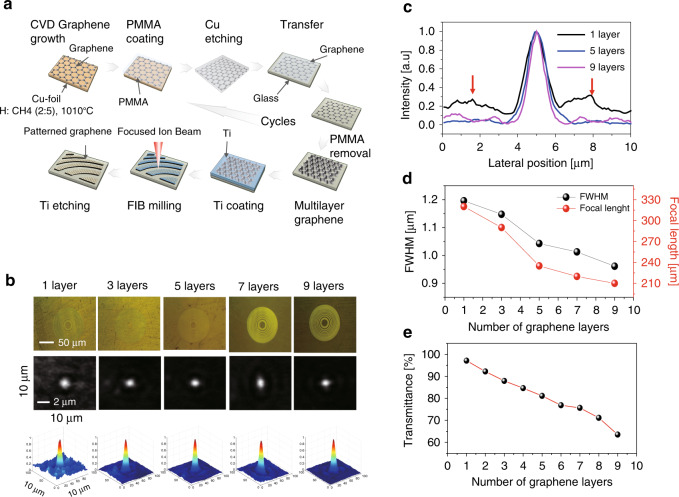
Optical characteristics of graphene FZP-based lenses depending on the number of graphene layers. **a** Fabrication of a graphene FZP-based lens array for a display pixel. The CVD-grown graphene was transferred onto a piece of glass using a PMMA coating, and a FIB was used for patterning the lens. The intensity distribution of our graphene FZP was examined for different numbers of layers, and the contrast and concentration are enhanced by increasing the number of graphene layers. **b** Optical images and 2D and 3D intensity distributions of graphene FZPs with 1, 3, 5, 7, and 9 layers. **c** Intensity distribution along the centre of the focal point detected at a focal length of 210 μm for the graphene FZP. **d** Trends of the FWHM and focal length with respect to the number of graphene layers, and **e** trend of transmittance values with respect to the number of graphene layers

A graphene-based single USSL was fabricated based on multilayer graphene and the FIB technique, as shown in Fig. 2a. A titanium coating was applied before FIB milling to improve the accuracy of the lithography. We used graphene obtained by chemical vapor deposition (CVD) at 1010 °C to fabricate graphene surfaces on a transparent glass substrate, using the wet transfer technique. Graphene stacks ranging from one to nine layers were investigated to study the variation in the transparency and focusing efficiency, with the number of graphene layers. The transmittance of graphene linearly decreases with increasing number of graphene layers; when the number of graphene layers is greater than five, the transmittance is reduced by >20% (Supplementary Fig. 4a, b). The graphene transferred onto the glass substrate was placed on the display of a Galaxy Note 8 (Samsung) mobile phone to examine the transmittance and the colour chromaticity characteristics. The colour chromaticity was measured with respect to the number of graphene layers, using a CS-2000 (Konica Minolta) spectroradiometer. According to the results, five-layer graphene was the most suitable graphene stack tested for display applications because the white chromaticity shift was less than the required *Wx* = 0.008 or *Wy* = 0.008 (Supplementary Fig. 4c, d). To further optimise the number of graphene layers, the focusing capability with respect to the number of layers of the graphene-based single USSL with the conventional ring pattern was also determined, as shown in Fig. 2b. An increase in the number of graphene layers enhanced the focusing characteristics (Fig. 2c), with the full-width at half-maximum (FWHM) decreasing (Fig. 2d) and the focal length converging to the designed value of 218 μm (Fig. 2d). Five-layer graphene was therefore chosen as the optimal single USSL based on our aforementioned results on the colour coordinates, transmittance (Fig. 2e), and focusing capability.

**Fig. 3:**
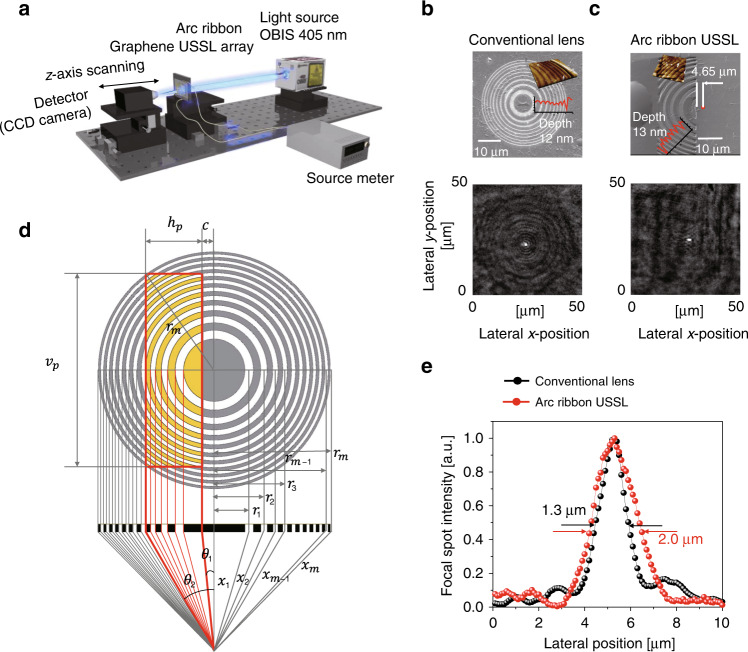
Design characteristics of the USSL. **a** Experimental setup for the transmittance-type Fresnel lens with a light source emitting at 405 nm, an arc ribbon graphene-based USSL array, and a CCD microscope. SEM and optical images of the **b** conventional FZP and **c** arc ribbon USSL. The insets show AFM results of our samples, which exhibit a clear distinction between the graphene and the open areas, with a depth of 12–13 nm. **d** Single USSL design based on the focal length. **e** FWHM comparison (conventional lens 1.3 μm, arc ribbon USSL 2.0 μm) of ring ribbon (black) and arc ribbon (red) lenses at the focal point

A graphene-based single USSL was fabricated, and the diffraction pattern was experimentally measured using a laser light source with a wavelength of 405 nm (OBIS 405LX/LS, Coherent), as shown in Fig. 3a. In the scanning electron microscopy (SEM) images shown in Fig. 3b, the dark grey colour represents the areas where the graphene has not been removed, and the bright grey colour represents the areas where the graphene has been removed by the FIB. Figure 3c presents a red pixel lens with design parameters *r*_1_ = 12.97 μm and *f* = 140 μm for 630 nm at *θ*_1_ = 2°, *θ*_2_ = 12°, *h*_*p*_ = 26 μm, and *v*_*p*_ = 78 μm. The thickness of the five-layer graphene was determined to be 12–13 nm based on atomic force microscopy (AFM) measurements, as shown in the SEM images in Fig. 3b, c (more information is provided in Supplementary Fig. 6). The focal spot position of the single USSL obtained by our simulation is the same as that of the conventional FZP lens, which indicates good consensus on the focal spot position of a single USSL for a multiview display between the simulated and experimental results in this study. At the focal length, the FWHM of the conventional FZP lens was 1.3 μm, and that of the USSL was 2.0 μm (Fig. 3e). The FWHM of the FZP lens increased with increasing *r*_1_ (ref. ^7^); however, the FWHM of the USSL was as low as 2.0 μm even for an *r*_1_ value of 12.97 μm. This difference in the FZP lens and the USSL indicates that the focusing efficiency of the USSL is remarkably high. In addition, the focusing efficiency is defined as the ratio of the intensity at the focal spot plane (*I*_*F*_) to the incident intensity transmitted through the focal objective plane (*I*_0_; Supplementary Fig. 6). Under the same focal length and standard FZP conditions, the circular lens showed 62.80% and the single subpixel square lens showed 62.79% focusing efficiencies. The subpixel square lens did not show a significant difference in focusing efficiency compared with the circular lens.

### Design characteristics of the USSL

**Fig. 4:**
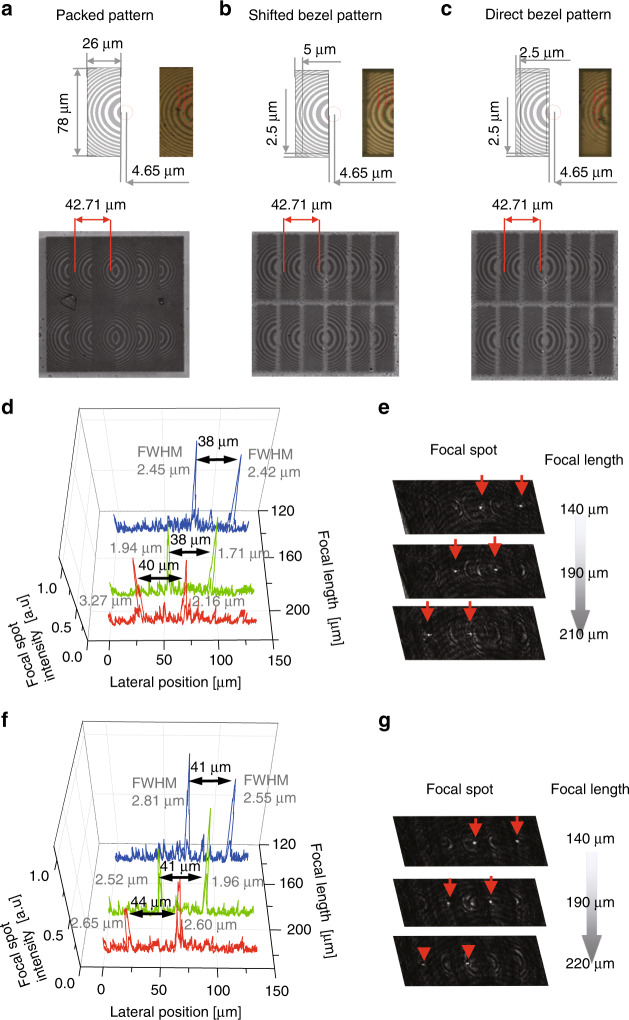
Focal length tuning by design of USSLs. Layout of the FIB treatment, and SEM images of the arc ribbon USSL arrays with designs of **a** a packed pattern, **b** a shifted bezel pattern, and **c** a direct bezel pattern. Distributions of the beam intensity and FWHM of the red USSL (red line), the green USSL (green line), and the blue USSL (blue line) in terms of the lateral position at the focal length for the **d** packed pattern and **f** shifted bezel pattern with the wavelength- (405 nm) dependent design. Variation in the focal length when the design is the **e** packed pattern (red USSL 210 μm, green USSL 190 μm, and blue USSL 140 μm) and **g** shifted bezel pattern (red USSL 220 μm, green USSL 190 μm, and blue USSL 140 μm)

Based on the results of the single USSL, we further investigated lenses with six subpixels (RGBRGB) to demonstrate the multifunctional capability of the USSL. The subpixels, which represent the minimum number of pixels (six subpixels) required to display an image in different directions from the display, were designed based on the pixel size (*h*_*p*_ = 26 μm, *v*_*p*_ = 78 μm; Supplementary Fig. 7). The USSLs correspond to each colour (red, green, and blue) of the display and were designed with radiuses *r*_1_, *r*_2_, *r*_3_,…, *r*_*m*_ and with *λ* = 630 nm (*m* = 13) for the red pixel lens, *λ* = 550 nm (*m* = 15) for the green pixel lens, and *λ* = 445 nm (*m* = 19) for the blue pixel lens (Supplementary Table 1). Consequently, different numbers of rings were used such that the subpixel lenses had the same focal length, while preventing chromatic aberration for each colour. In the colour filter of the display device, a black matrix (BM) was inserted between different colours to prevent possible light leakage in the bus line of the thin-film transistor and colour mixture due to the FOV. The BM in the display device changes the radius *r*_1_ of the pixel lens, resulting in a change in the focal length due to diffraction. We analysed three ETF-USSL designs for multifunctional autostereoscopic displays. In the first design, the ETF-USSL pattern fully filled the pixel pitch (Fig. 4a), whereas in the second design, a cut shifted by a bezel width of 2.5 μm toward the centre of the rings of the USSL was employed (Fig. 4b), and in the third design, the cut was directly matched with the bezel width of 2.5 μm at the original position (Fig. 4c).

The distributions of the focal lengths of the three designs of the ETF-USSL were measured using a 405 nm laser source. Among the three designs, the packed and shifted bezel patterns showed high focal spot intensity and FWHM, and the focal spot changed from the blue USSL spot to the red USSL spot with increasing focal length, as shown in Fig. 4d–g. Although our USSLs were designed to achieve the same focal length for the three wavelengths of the display device, three focal spots were observed because only one light source with a wavelength of 405 nm was used. Despite only using a 405 nm light source, the results agreed well with the theoretical estimations. The designed focal spot distance between the left square subpixel lens (LSSL) and the right square subpixel lens (RSSL) was 42.71 μm, with values of 38–40 μm obtained for the packed pattern (Fig. 4d) and 41–44 μm for the shifted pattern (Fig. 4f), which are close to the designed value. This result demonstrates that our graphene-based USSL can realise multifocusing using the same plane lens. The focal lengths of the packed pattern were 140 μm for the blue USSL, 190 μm for the green USSL, and 210 μm for the red USSL when using the 405 nm light source. These are slightly smaller than the simulation results for the USSL (focal lengths of red USSL 218 μm, green USSL 190 μm, and blue USSL 154 μm), but the values are close (Fig. 4e). A similar trend was observed for the shifted bezel pattern design (Fig. 4f). However, the focus of the direct bezel pattern design was unclear in the entire focal spot at the focal plane (Supplementary Fig. 8). Table 1 presents a summary of the focal length, focal spot distance, and focusing efficiency of the packed and shifted bezel pattern designs. Overall, the focusing efficiencies were the highest for the packed design, >60% for the red, green, and blue lenses. The results indicate the following: the focal length of the USSL depends on the size of the first ring; the focusing efficiency depends on the number of rings; and the focal spot position depends on the distance from the centre of the semicircular rings. The characteristics of the focal spots of the packed pattern and the shifted bezel pattern, whose ring size was not reduced by the bezel, were therefore excellent, and the packed pattern with the largest number of rings had the highest focusing efficiency.

**Table 1:** Focusing characteristics of USSL designs

USSL	Focal length (μm)			Focal spot distance (μm) (LSSL–RSSL)				Focusing efficiency (%)
	Design	Result		Design	Result		Result	
		Packed pattern	Shifted bezel pattern		Packed pattern	Shifted bezel pattern	Packed pattern	Shifted bezel pattern
Red	218	220	210	42.71	38	41	61.18	60.61
Green	190	190	190	42.71	38	41	60.96	58.29
Blue	154	140	140	42.71	40	44	64.92	62.98

Design parameters, variations in the focal length and the focal spot distance between the LSSL and RSSL, and focusing efficiency at the focal length for the wavelength-dependent design (Fig. 4) of the arc ribbon USSL array based on the packed pattern and shifted bezel pattern

### Tuneable focal length of the ultrathin subpixel lens

**Fig. 5:**
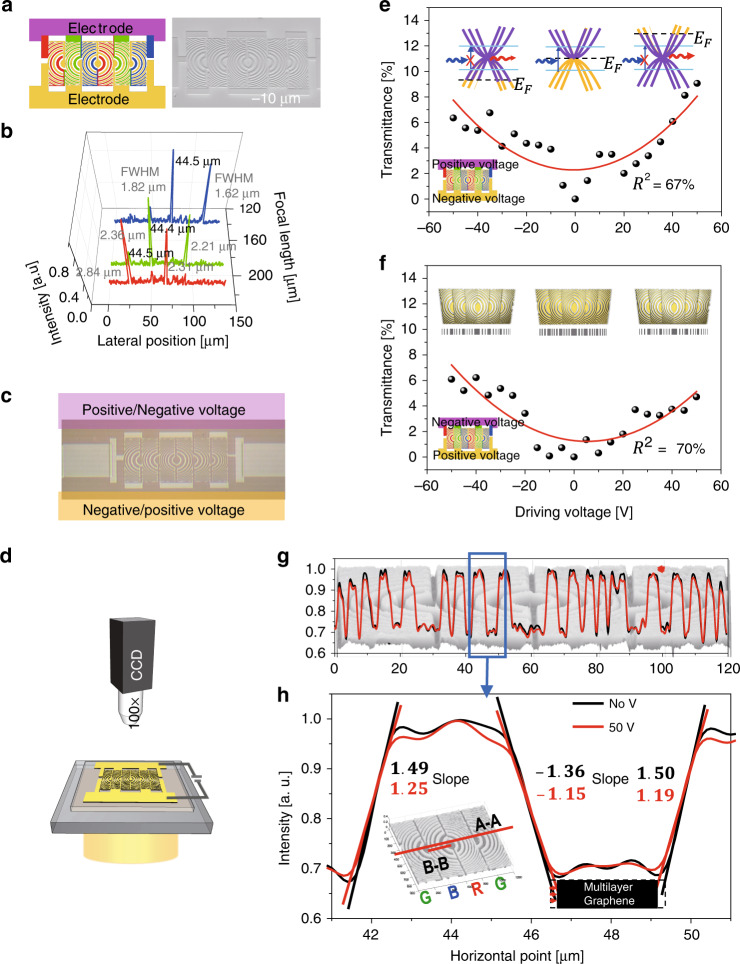
Transmittance and intensity profile for the tuneable focal length ETF-USSL. **a** Layout of the FIB treatment, and SEM image of the USSL array with additional electrodes (magenta and yellow) based on the packed pattern. **b** Variation in the focal length and distribution of beam intensities (FWHM of red USSL 2.84 μm/2.31 μm, FWHM of green USSL 2.36 μm/2.21 μm, and FWHM of blue USSL 1.82 μm/1.62 μm) at the focal length (210 μm/190 μm/140 μm for the red/green/blue USSL, respectively) for the wavelength-dependent design (218 μm/190 μm/154 μm for the red/green/blue USSL, respectively). **c** Optical image of the USSL array with electrodes (magenta and yellow) for application of a DC voltage bias (positive and negative voltage) for focal length modification by an electric potential. The 2D and 3D intensity distributions of the USSL were measured by **d** a 100× optical microscope under an applied DC voltage bias. Experimental results of **e** the transmittance when positive voltages are applied to the upper electrode (magenta), and negative voltages are applied to the lower electrode (yellow) and **f** vice versa. The coefficients of determination (*R*^2^) of the red curves in **e** and **f** are 67 and 70%, respectively. The transmittance of the USSL is the lowest near 0 V, but much greater at −50 V or +50 V. Difference in the absolute slopes of the intensity profile with an applied DC voltage bias (no voltage (black line) and 50 V (red line)), resulting from the size effect of the arc ribbon due to light transmission for the USSL, in **g** section A–A, which consists of green–blue–red–green USSLs with a length of 120 μm, and **h** section B–B with a length of 10 μm. The absolute slopes of section B–B are shown in **h**. The absolute slopes are low, and the edge intensities of the multilayer graphene pattern are high in the enlarged area

To control the focal length of the graphene-based USSL, we designed additional electrodes based on the packed pattern (Fig. 4a), as shown in Fig. 5a. The electrical wire for connecting USSL arc ribbons was 1 µm in width, and the distance between each arc ribbon was 1.5 µm in length. The advantage of the packed pattern design is that the connection wires are extensions of the initial arc ribbons in the pattern, and thus do not affect the initial USSL pattern or its efficiency (Supplementary Fig. 9). The focal length values of the USSL with an electrode (Fig. 5b, Table 2) were similar to the designed values of 218 μm, 190 μm, and 154 μm for red, green, and blue, respectively, which were achieved by optimising the FIB milling conditions (Crossbeam 540, Zeiss, Ga ion source, 30 kV/3 nA milling current, 0.8 dose factor). Furthermore, the focal spot distance in the lateral direction was ~44.5 μm, which well matched the designed value of 44.7 μm, with a focusing efficiency over 60% (Table 2). The focusing characteristics of the USSL are demonstrated in Supplementary Video 1, which shows the focal spot position moving from the blue USSL spot at the right position to the red USSL spot at the left position, with increasing focal length. Supplementary Video 2 shows the focal spot intensity in Supplementary Video 1, represented in three dimensions using a MATLAB simulation.

**Table 2:** Tuneable focusing characteristics of the USSL

	Design			Experimental results			
USSL	Focal spot distance (LSSL–RSSL) (*d*_*f*_, μm)			Focal spot position shift (Δ*c*, μm)	Focal length shift (Δ*f*, μm)	Focal length (*f*, μm)	Focusing efficiency (%)
		0 V	50 V				
Red	44.7	44.5	45.5	0.50	26.2	210	62.79
Green	44.7	44.4	45.9	0.75	36.9	190	60.11
Blue	44.7	44.5	45.4	0.45	16.4	150	64.84

Focal length, focusing efficiency, and focal spot distance variation, focal spot position shift and focal length shift at the focal plane of the ETF-USSL with an applied voltage for the wavelength-dependent design of the arc ribbon USSL array with additional electrodes based on the packed pattern (Fig. 5)

To apply a horizontal fringe field to the USSLs, the arc ribbon patterns of red, green, and blue colours in each USSL subpixel were connected to a relatively large pad, indicated by magenta in Fig. 5a, to ensure that a DC voltage bias could be applied (Fig. 1)^42^. A driving voltage bias was applied from −50 V to +50 V in steps of 5 V. Figure 5e shows the transmittance results obtained with a transmission microscope using a lens with a magnification of 100× (Fig. 5d), when positive voltages were applied to the upper electrode and negative voltages were applied to the lower electrode. Meanwhile, Fig. 5f shows the transmittance results when voltages with opposite signs were applied to the electrodes. The results showed that the transmittance of the USSL is the lowest near 0 V and highest at −50 V or +50 V. These results were the same regardless of whether the driving voltage was applied to the even (Fig. 5e) or odd (Fig. 5f) patterns in the USSL. Consequently, the transmittance was improved by up to 9% at 50 V compared with that at 0 V. This increase in transmittance under a driving voltage can be explained using the intensity profile of the ETF-USSL. Figure 5g shows the intensity profile in section A–A, which consists of green–blue–red–green USSLs with a length of 120 μm, under both a 50 V driving voltage and no voltage (0 V). Compared with that at 0 V, the absolute slope for section B–B at 50 V is low, as shown in Fig. 5h, and the edge intensity of the multilayer graphene pattern is high in the enlarged area. When the in-plane electric field generated by the DC voltage bias relatively shifts the carrier density to the left and right sides of the graphene-based arc ribbon, the transmittance at the edge increases because the Fermi level of the graphene is far from the Dirac point (Fig. 1d). This has the same effect as electric reduction of the size of the arc ribbon USSL. Ultimately, the reduction of the size of the arc ribbon indicates that the focal length can be increased by the improved linearity of the diffraction by the ETF-USSL.

**Fig. 6:**
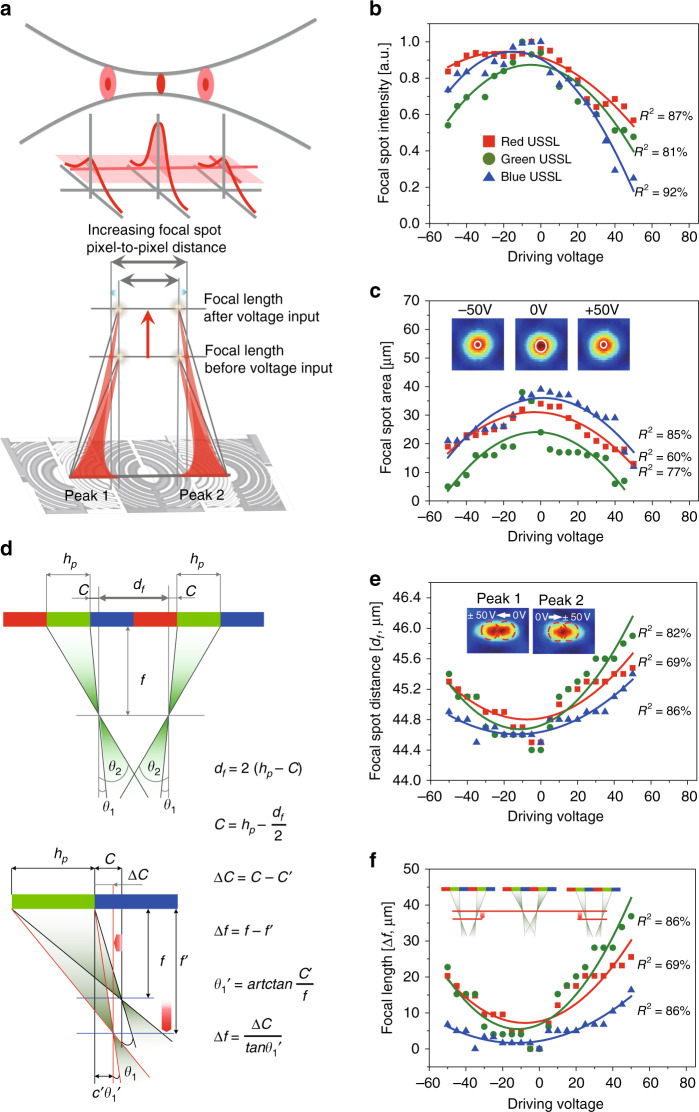
Focal spot characteristics with focal length variation of the ETF-USSL. Schematics of the focal point shift with application of an electric field: **a** the spot intensity depends on the distance along the *z*-axis and the distance in the lateral direction between the focal spots of peaks 1 and 2. At a fixed focal length position, the maximum intensity of the focal spot decreases as the focal length of the USSL becomes longer owing to the driving voltage. Experimental results of **b** the intensity variation at the focal point and **c** the spot size. As the driving voltage increases or decreases from 0 V to ±50 V, the focal length increases and the intensity of the focal spot decreases; thus, the spot size with maximum intensity also decreases. The images in graph **c** show that the maximum intensity is lower at ±50 V than at 0 V. **d** Formula for calculating the focal length (Δ*f*) by varying the focal spot distance (*d*_*f*_). **e** The focal spot distance (*d*_*f*_) is the distance between the two peaks at the focal length, with the lowest distance near 0 V, which gradually increases for positive and negative voltages. The peak 1 and peak 2 images on the graph show the behaviour of the focal spots of the left and right green USSLs, when the driving voltage increases from 0 V to ±50 V. The peak 1 focal spot moves from right to left, and peak 2 moves from left to right. The focal spot distance (*d*_*f*_) at a fixed focal plane increases when the focal length of the ETF-USSL increases. **f** The focal length shift (Δ*f*) is calculated based on the focal spot distance. As the voltage increases or decreases from 0 V, the focal length increases, with the change in the green focal length the largest among the three. Eventually, the focal length changes as shown on the graph, indicating changes in the FOV. In **b**, **c**, **e**, and **f**, *R*^2^ is the coefficient of determination for the red, green, and blue curves

Figure 6 also validates the shift of the focal point of the ETF-USSL with the driving voltage. As the focal length varies due to the variation in the voltage, the maximum intensity decreases because the focal spot intensity at a fixed focal length decreases. Figure 6a illustrates this relation. As the focal length increases, the maximum intensity in the fixed focal plane decreases, which reduces the area of focal spots of the same intensity and increases the focal spot distance (*d*_*f*_) between pixels. The maximum intensity is the highest ~0 V, and decreases in the positive and negative voltage directions in Fig. 6b, also indicating the focal length shift. Furthermore, all red, green, and blue USSLs showed the same characteristics. Under realistic conditions, the graphs were not symmetric; the transition was widened by the graphene defects, with a shift toward lower voltages from 0 V due to natural doping from the glass substrate^43^. In these circumstances, the focal spot area is the largest at ~0 V and decreases at other voltages, as shown in Fig. 6c, which is due to a shift in the focal length. On the graph in Fig. 6c, the two-dimensional (2D) images of the lateral directions of the focal spot show that the deep red area at 0 V did not appear at −50 V and +50 V. Here, the focal spot distance (*d*_*f*_) decreases when the external potential reaches 0 V in Fig. 6e. The peak images on the graph show the behaviour of the focal spots of the left and right green USSLs, as the driving voltage increase from 0 V to ±50 V. The peak 1 focal spot moves from right to left, and peak 2 moves from left to right on a fixed focal plane. This means that the focal length becomes longer. The focal length shift (Δ*f*) can be obtained by calculations based on the measured focal spot distances (*d*_*f*_) and focal spot positions (*c*; Fig. 6d). The calculations show that the focal lengths of the lenses for the three pixels increase when the voltage is positive or negative, as shown in Fig. 6f. In summary, the focal spot intensity, focal spot area, and focal spot distance all clearly demonstrate the focal length shift.

As a result (Table 2), the focal spot position shifts (Δ*c*) of the two peaks moved outward with increasing voltage, and the focal position shift in a fixed focal plane showed an increase in the focal length; the red focal spot was shifted by 25.6 μm, the green focal spot was shifted by 36.9 μm, and the blue focal spot was shifted by 16.4 μm at a potential of 50 V. The largest change, observed for the green focal spot, is due to the largest voltage effect. Because the green USSL is between the red and blue USSLs, the first red USSL is open on the left, and the last blue USSL is open on the right. This explains why the ETF-USSL focal length of 190 μm can vary up to 19.42% (36.9 μm) after applying a voltage. This indicates that the FOV of the observer can also be controlled by the electromagnetic field in the ETF-USSL in multifocusing.

## Discussion

The graphene-based ETF-USSL with five graphene layers had a transmittance of 82% and a focusing efficiency of >60%. In our lenses with the arc ribbon design, the effective spacing of the arc ribbons varies due to the difference in the carrier distribution depending on the position of the electric field. This results in a variation of the diffraction characteristics of the slit such that the focal length can be adjusted in the visible regime without any change in the design. The ETF-USSL can be customised according to the wavelength of each subpixel in the display device without any additional light source^5^ or device. The ETF parameters are shown in Table 2. The 19.42% change in focal length is due to the transmission of light under the driving voltage. In this case, the graphene arc ribbon is crucial in changing the focal length, resulting in further important electrical and optical properties. Our ETF-USSL for display circuits can support multifunctional autostereoscopic applications based on an ultrathin device. By exploiting the advantages of its structure at the subpixel scale, the ETF-USSL mechanism can be embedded into each individual pixel in glassless 3D displays, privacy displays, and multiview displays for display applications. In addition, this ETF-USSL design can be customised for 3D hologram displays^44^, acoustic applications^45^, and optical devices comprising metasurfaces^46,47^.

## Materials and methods

### CVD synthesis of graphene

For single-layer graphene, a Cu substrate was first annealed at 1010 °C for 30 min, and single-layer graphene was grown in a chamber at 1010 °C with methane (with a CH_4_ gas partial pressure of 290 mTorr and a corresponding flow rate of 20 ccm) and hydrogen (with a H_2_ gas partial pressure of 100 mTorr and a corresponding flow rate of 8 ccm). The growth procedure was performed using a commercially available 100-μm-thick Goodfellow Cu foil (item #CU000565) with a diameter of 50 mm as the growth substrate in a quartz chamber. The graphene grown on the Cu foil was spin-coated with a poly(methyl methacrylate) (PMMA) layer (PMMA 950 K A6, MicroChem Corp.) for 32 s at 4200 rpm to form a supporting layer, which was baked at 180 °C for 2 min to improve the adhesion to the substrate.

### Transfer of graphene

To remove the Cu substrate from the PMMA/graphene/Cu stack layer, the layer was transferred onto glass after wet etching and washing with water. For wet etching, we used an aqueous solution of ammonium persulfate ((NH_4_)_2_S_2_O_8_, Sigma-Aldrich). Finally, after air-drying, this process was repeated five times to produce five graphene layers (Fig. 4).

### Fabrication of USSLs

USSLs were patterned using the FIB technique. To pattern graphene, titanium was deposited onto the graphene layer and subjected to FIB milling (Crossbeam 540, Ga ion source, 30 kV/3 nA milling current, 0.8 dose factor). Oxygen plasma was used to remove titanium from the regions where it was milled by the FIB. The titanium on the remaining graphene was removed, using a titanium etchant (Fig. 2a).

### Optical characterisation of the graphene layer

An ultraviolet/visible spectrometer (V-650, JASCO Corp.) was used to measure the transmittance in the wavelength range of 200–900 nm (Supplementary Fig. 5a, b). The spectrum intensity and colour chromaticity were measured on a Galaxy Note 8 mobile phone with respect to the number of graphene layers using a spectroradiometer (CS-2000, Konica Minolta; Supplementary Fig. 5c, d). The spectrum intensity was measured in the range of 300–900 nm.

### Focusing characterisation of the ETF-USSL

The focusing performance of the ETF-USSL was examined by measuring the light intensity of a focal spot through a microscope with a magnification of 40. A 405 nm light beam from a laser (OBIS 405–200 C, Coherent) illuminated the lens, and its focal response was measured with a charge-coupled device (CCD) camera. The DC voltage bias of the ETF-USSL was measured using a source measure unit (2612 SourceMeter, Keithley) at 1 min intervals, from −50 V to +50 V in steps of 5 V (Figs. 5 and 6). The first step was to measure the positive voltage from 0 V to 50 V and back to 0 V, and then the negative voltage from 0 V to −50 V to confirm that the same characteristics could be obtained when returning to 0 V, as the increase in electrical stress could be attributable to other factors. To determine the focusing characteristics of the USSL, the focal spot intensities in the CCD camera images were obtained by image processing using MATLAB software.

## Supplementary information

Supplementary information for Electrically focus-tuneable ultrathin lens for high-resolution square subpixels

Supplementary Video 1

Supplementary Video 2
