# Supplementary material for: Electrically focus-tuneable ultrathin lens for high-resolution square subpixels
Source: Light Sci Appl. 2020 Jun 5;9:98. doi: 10.1038/s41377-020-0329-5 (PMC7275053; doi:10.1038/s41377-020-0329-5)
Supplement: Supplementary file 1 — Supplementary information for Electrically focus-tuneable ultrathin lens for high-resolution square subpixels [file 41377_2020_329_MOESM1_ESM.docx]

**Supplementary information**

**Electrically focus-tuneable ultrathin lens for high-resolution square subpixels**

Sehong Park^1^, Gilho Lee^1^, Byeongho Park^1^, Youngho Seo^1^, Chae bin Park^1^, Young Tea Chun^2,3^, Chulmin Joo^1^, Junsuk Rho^4,5,6^, Jong Min Kim^2^, James Hone^7^, Seong Chan Jun^1*^

^1^School of Mechanical Engineering, Yonsei University, 50 Yonsei-ro, Seodaemun-gu, Seoul 03722, Republic of Korea

^2^Electrical Engineering Division, Engineering Department, University of Cambridge, 9 JJ Thomson Avenue, Cambridge, CB3 OFA, United Kingdom

^3^Department of Electronic Material Engineering, Korea Maritime and Ocean University, Busan 49112, Republic of Korea

^4^Department of Mechanical Engineering, Pohang University of Science and Technology (POSTECH), 77 Cheongam-ro, Nam-gu, Pohang 37673, Republic of Korea

^5^Department of Chemical Engineering, Pohang University of Science and Technology (POSTECH), 77 Cheongam-ro, Nam-gu, Pohang 37673, Republic of Korea

^6^Institute for Convergence Research and Education in Advanced Technology, Yonsei University, 50 Yonsei-ro, Seodaemun-gu, Seoul 03722, Republic of Korea

^7^Department of Mechanical Engineering, Columbia University, 500 West 120th Street, Mudd 220, New York, NY 10027, USA

Keywords: Electrically tuneable focusing, Square subpixel lens, Graphene, Flat lens, Fermi level, Fresnel zone plate, Multifunctional display

^*^Corresponding author

Seong Chan Jun

Tel: +82-2-2123-5817; E-mail: scj@yonsei.ac.kr


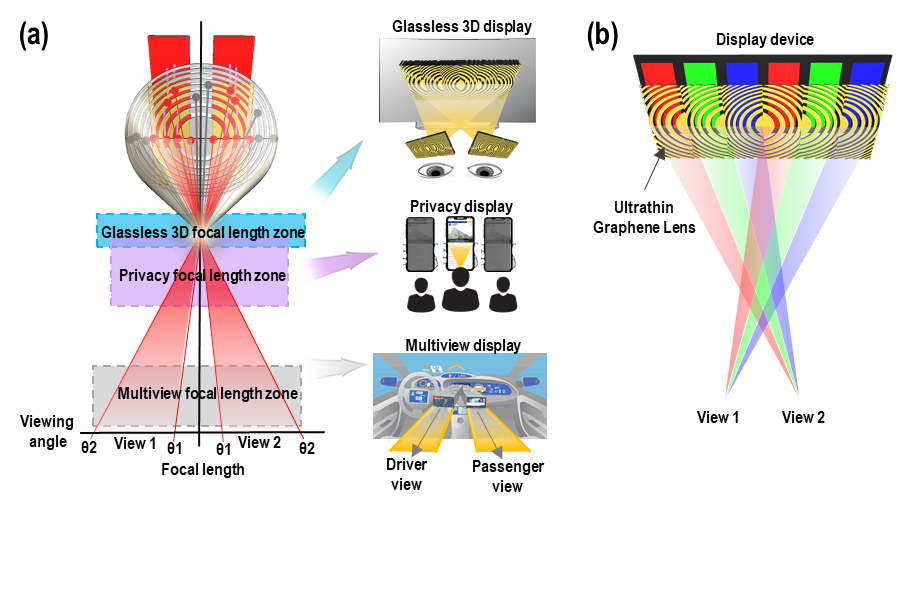


**Supplementary Figure S1. Display applications based on focal length variation of the USSL.** (a) Schematic of a conventional multifunctional subpixel lens based on Fresnel lenses. As the focal length increases, stereoscopic display of glassless 3D, privacy function, and multiview function can be implemented. (b) Specific ultrathin graphene lens for red–green–blue/red–green–blue (RGB/RGB) subpixel on a display device. An RGB subpixel for view 1 on the right side (or the right eye) and neighboring pixels for view 2 on the left side (or the left eye) are designed that can be fitted in the pixel structure with a Fresnel lens pattern.


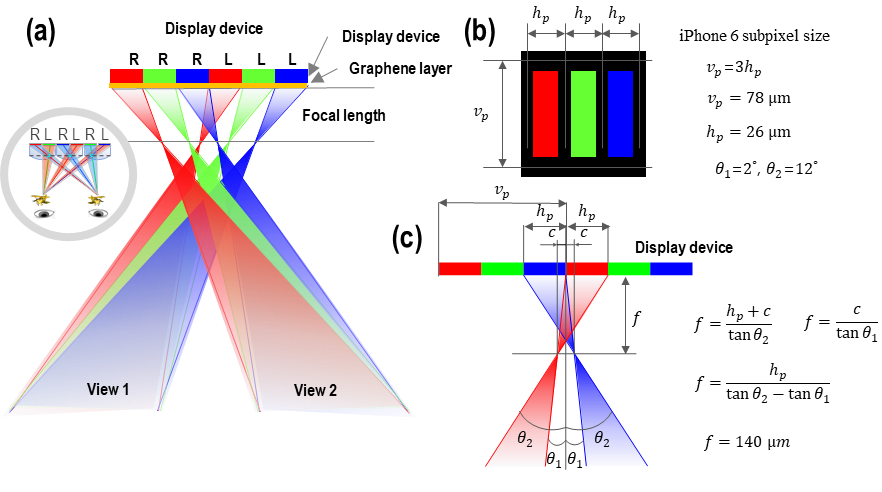


**Supplementary Figure S2. Focal length design for multifunctional subpixel lens.** (a) Distribution of the viewing zone, (b) subpixel of the display device, and (c) viewing parameters and subpixel dimension.

**
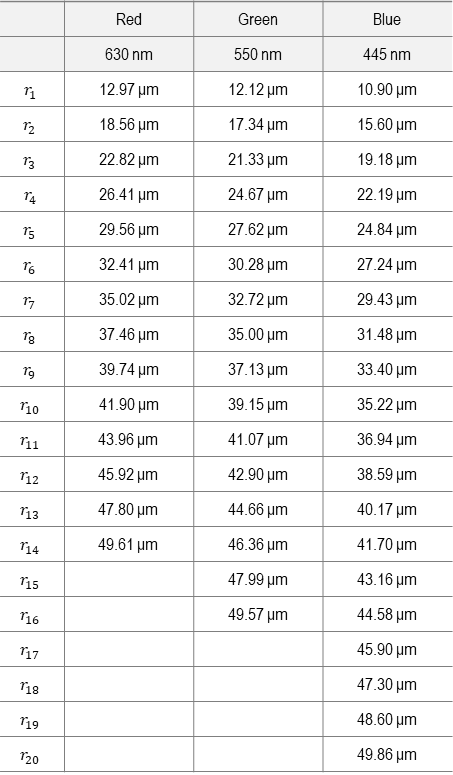
**

**Supplementary Table S1. Design parameters of the USSL.** Design parameters of the USSL based on the focal length for the wavelength of each subpixel.


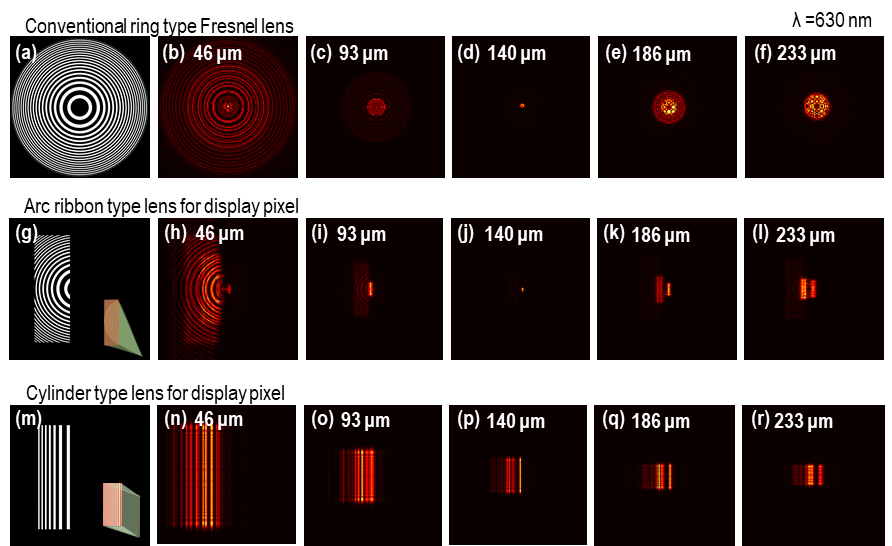


**Supplementary Figure S3.** **Simulated result of a single USSL.** Simulated result of diffracted patterns using the Rayleigh–Sommerfeld theory for three different types of single-layer graphene FZP under an incident beam of 630 nm. (a)–(f) Conventional ring-type Fresnel lens (radius of 49.61 μm) (λ = 630 nm, *r*_1_ =12.97 μm, and *f* =140 μm), (g)–(l) arc ribbon type lens for subpixel of display (26 × 78 μm) (*θ*_1_ *=* 2°, *θ*_2_ *=* 12°, *h*_p_ = 26 μm, and *v*_p_ =78 μm), and (m)–(r) cylinder type lens for subpixel of display (26 × 78 μm) within focal region of 0.00–233 μm along the beam path.


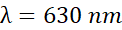

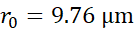

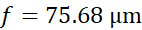

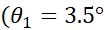

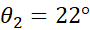

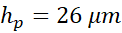

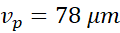


**
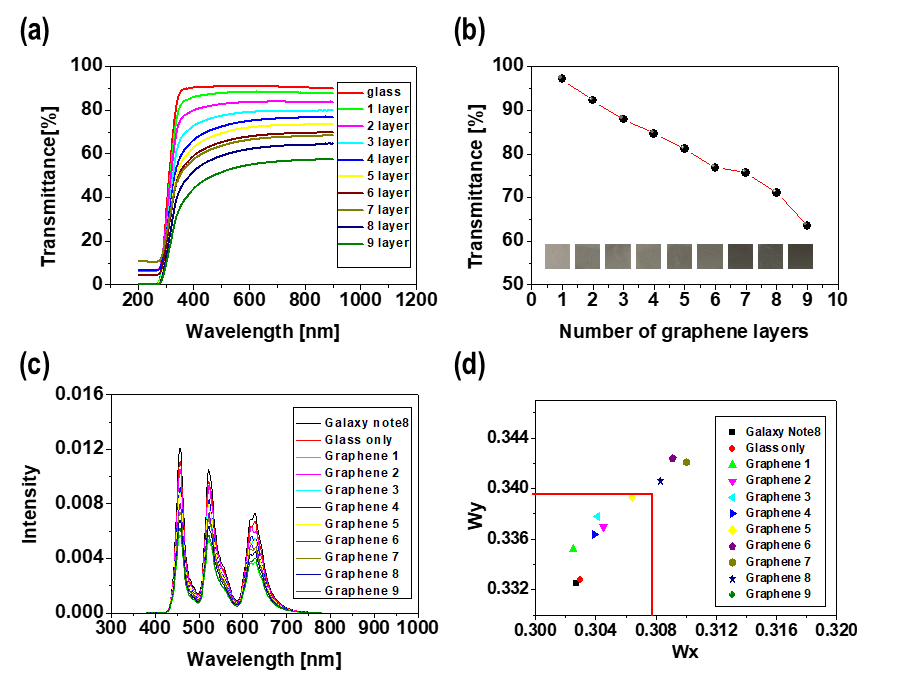
**

**Supplementary Figure S4. Dependency of transmittance on the number of graphene layers.** (a) Transmittance spectrum, (b) integrated transmittance in the range of 380–780 nm, (c) transmittance intensity under organic light-emitting diode (OLED) incident light from the display of a Galaxy Note 8 mobile phone, and (d) colour chromatic (CIE 1931) graph for several graphene sheets. Maximum-allowed-colour-difference region of the covered material sample is the limit for human eye perception (*W*: colour chromatic value, *ΔW_xy_* < 8/1000, ΔW = transmittance ratio of the intrinsic display and the material covered sample).


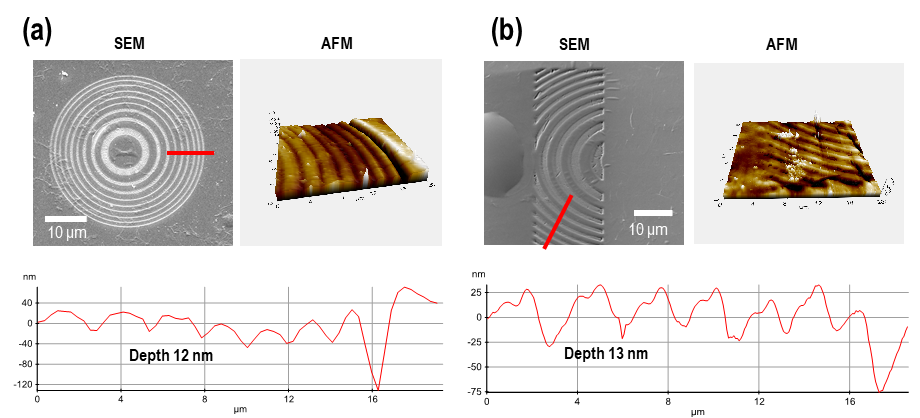


**Supplementary Figure S5. Characterization of the depth of the lens with five layers of graphene.** (a) Conventional FZP and (b) arc ribbon USSL. The insets show the SEM and AFM results of our samples. The result shows that the conventional FZP has a depth of 12 nm and the arc ribbon USSL has a depth of 13 nm.


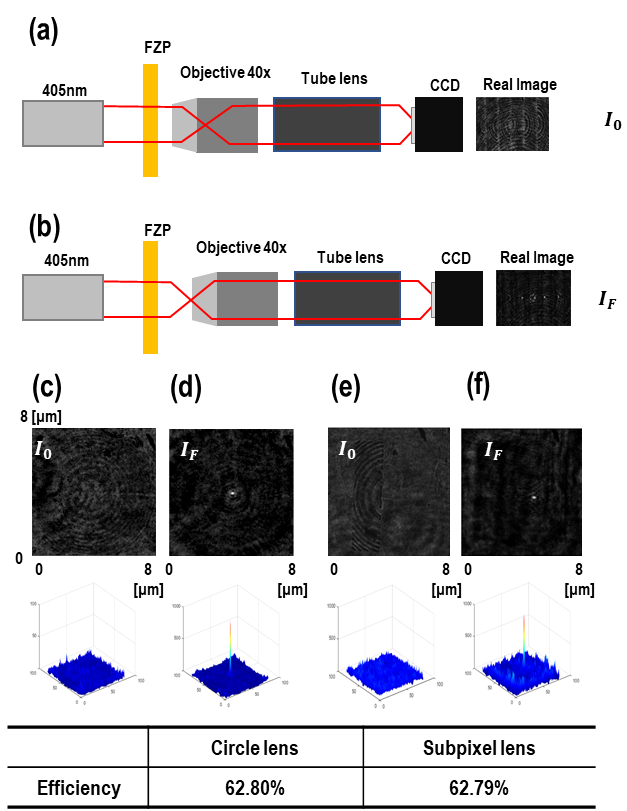


**Supplementary Figure S6. Characterization of focus efficiency of the FZP.** Focusing efficiency is defined as the ratio of (a) the intensity at the focal spot plane ($I_{F})$ to (b) the incident intensity transmitted through the focal objective plane ($I_{0})$. Illustration of the optical measurements at the focal objective plane ((c) for circle, (e)) for single subpixel) and at the focal spot plane ((d) for circle, (f) for single subpixel), followed by the intensity analysis of each image.


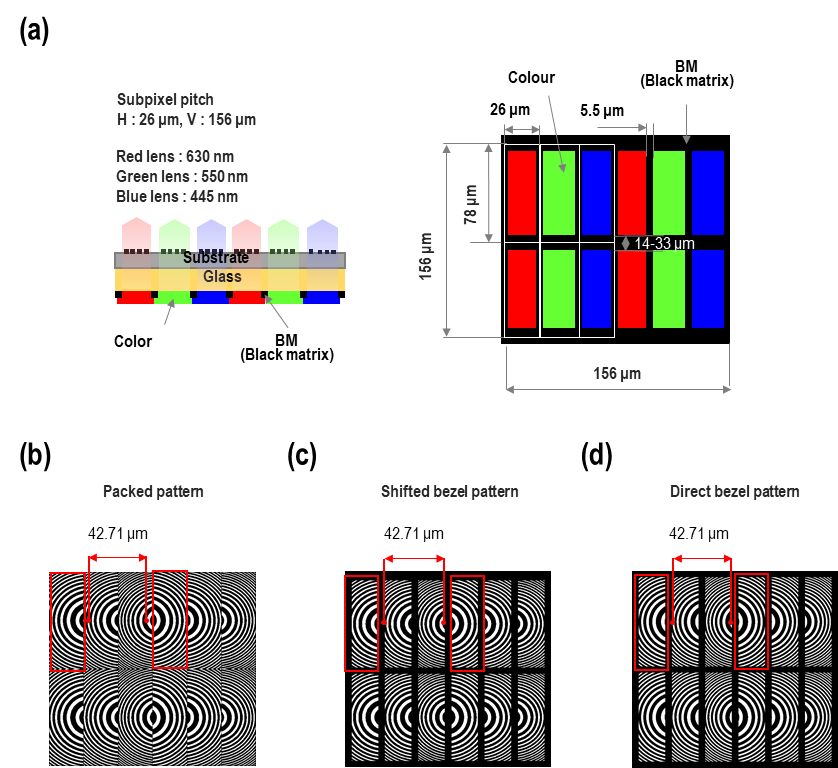


**Supplementary Figure S7. Three USSL designs.** (a) Specific design and layout of our graphene USSL array for an RGB pixel. (b) To compare the efficiency of among the patterns under consideration, three different patterns were designed. ($h_{p}=26 \mu m$ and $v_{p}=78 \mu m$; hence, 6 × 2 subpixels with $h_{p}=v_{p}=156 \mu m$).


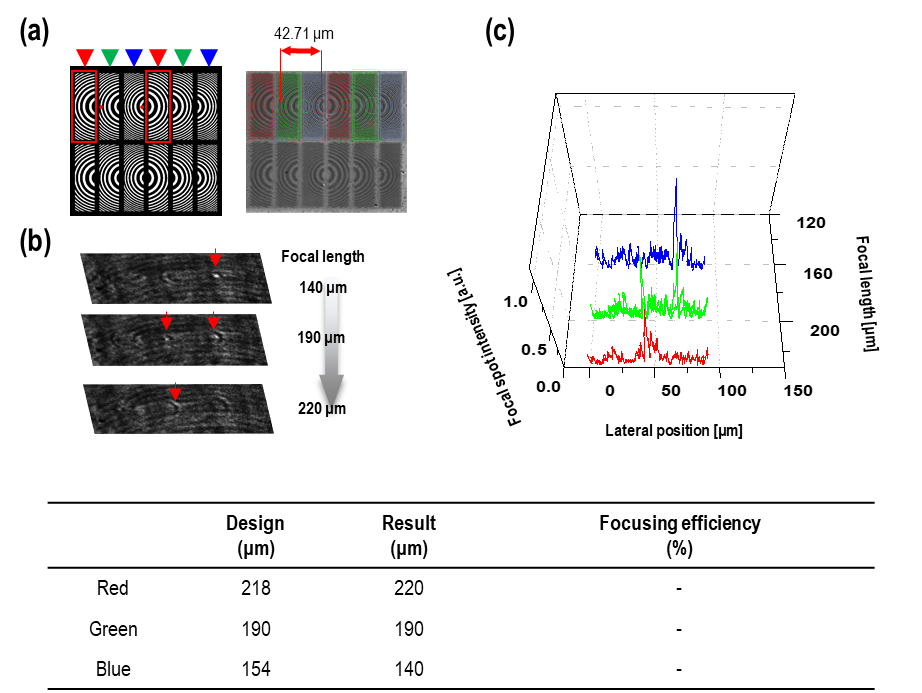


**Supplementary Figure S8.** **Focusing characteristics of the direct bezel design.** (a) Layout for the FIB treatment and the SEM image of arc ribbon USSL array with direct bezel pattern, (b) variation of focal length, and (c) distribution of beam intensity at the focal length with wavelength-dependent design.


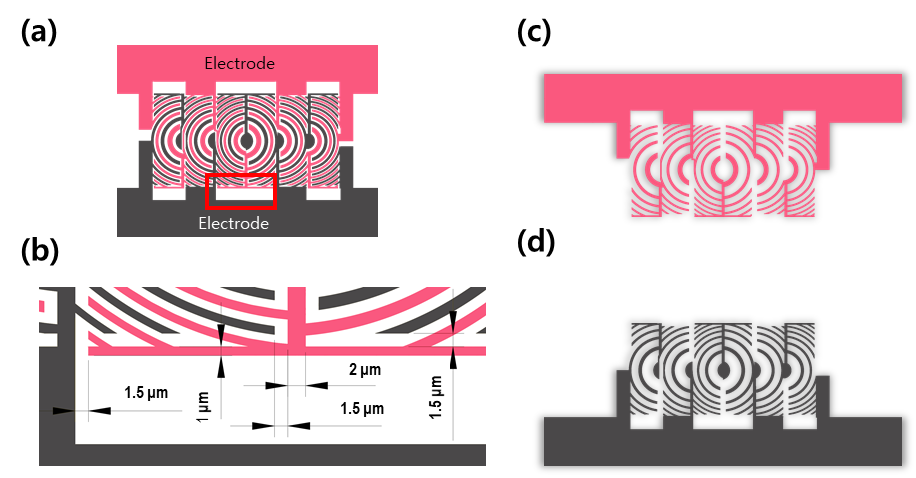


**Supplementary Figure S9.** **Design for applying DC-field to USSL**. (a) Each arc ribbon is connected to either pink or grey electrode in consecutive order. (b) The electrical wire for connecting USSL arc ribbons is 1um in width, and the distance between each arc ribbon is 1.5um in length. (c) The arc ribbons connected to the top electrode (pink). (d) the arc ribbons connected to the bottom electrode (grey).

**Supplementary Note 1**

**Fundamental formulas for the graphene-based square subpixel lens**

The Fresnel zone plate (FZP) is a circular diffraction grating with a radially increasing line density. The solution of the differential wave equation is^1^

|  | $E\left( x,t \right)=E_{0}\sin[\omega t-(kx+\varepsilon)]$. | (1) |
| --- | --- | --- |

By expressing the amplitude of the harmonic waves as

|  | $\alpha\left( x,\varepsilon\right)=-(kx+\varepsilon$), | (2) |
| --- | --- | --- |

we obtain

|  | $E\left( x,t \right)=E_{0}\sin[\omega t+\alpha\left( x,\varepsilon\right)$]. | (3) |
| --- | --- | --- |

Let us assume that two waves propagating at the same frequency and speed are superimposed in space as

|  | $E_{1}=$ $E_{01}\sin\left( \omega t+\alpha_{1} \right)$, | (4) |
| --- | --- | --- |
|  | $E_{2}=$ $E_{02}\sin\left( \omega t+\alpha_{2} \right)$. | (5) |

The superposition of waves is the linear sum of the two waves:

|  | ${E=E}_{1}+E_{2}$. | (6) |
| --- | --- | --- |

The resultant flux density of the synthesized wave is not added to the linear flux density of the individual component waves, but the term $2E_{01}E_{02}\cos(\alpha_{2}-\alpha_{1}$), known as the interference term, is added. The phase difference $\delta$ $\equiv$ $(\alpha_{2}-\alpha_{1})$ of the two interfering waves is an important factor:

|  | $E_{0}^{2}=E_{01}^{2}+E_{02}^{2}+2E_{01}E_{02}\cos(\alpha_{2}-\alpha_{1}$). | (7) |
| --- | --- | --- |

Considering that the phase difference is determined not only by the difference in the initial phase angle of the two waves but also by the difference in the paths through the two waves, we can write:

|  | $\delta=\left( {kx}_{1}+\varepsilon_{1} \right)-\left( {kx}_{2}+\varepsilon_{2} \right)$, | (8) |
| --- | --- | --- |
|  | $\delta=k\left( x_{1}-x_{2} \right)+\left( \varepsilon_{1}-\varepsilon_{2} \right)$. | (9) |

The relative the optical path length difference is $\Lambda=\Delta nt$, and as $\Delta\varphi=k\Lambda$ and the propagation number $k=2\pi/\lambda$, the phase difference is $\Delta\varphi=2\pi t\Delta n/\lambda$ when considering the factor of the initial phase shift ($\varepsilon_{1}-\varepsilon_{2}$) of the harmonic wave, where $\Delta n$ is the reflective index difference and *t* is the thickness of a single USSL:

|  | $\delta=\frac{2\pi}{\lambda}\left( x_{1}-x_{2} \right)+\Delta\varphi.$ |  | (10) |
| --- | --- | --- | --- |

When the phase shift $\delta$ = 2$m\pi$, the composite wave amplitude is maximal, $x_{2}=f$ and

|  | $\left( x_{m}-f \right)=m\lambda-\lambda\frac{\Delta\varphi}{2\pi} .$ | (11) |
| --- | --- | --- |

The distance $x$ between the source point and the focus, known as the optical path length, is given by:

|  | $x_{m}^{2}=r_{m}^{2}+f^{2}$. | (12) |
| --- | --- | --- |

Solving equation (12) for $r_{m}$ gives^2^

|  | $r_{m}=\sqrt{\left( m\lambda-\lambda\frac{\Delta\varphi}{2\pi} \right)^{2}+2f\left( m\lambda-\lambda\frac{\Delta\varphi}{2\pi} \right)},$  $\approx\sqrt{2\lambda f\left( m-\frac{\Delta\varphi}{\pi} \right)} m\lambda\ll f .$ | (13) |
| --- | --- | --- |

The maximum value of $r_{m}$ can be obtained from the subpixel size of the display:

|  | $r_{m\_max}=\sqrt{{( h_{p}+c )}^{2}+\left( \frac{v_{p}}{2} \right)^{2}}.$ | (14) |
| --- | --- | --- |

Therefore, the focal length can be calculated using the viewing angle of the view point and the horizontal subpixel size $h_{p}$. This can be used to design symmetrical FZP lenses, and the maximum size is related to $r_{m\_max}$. Supplementary Fig. 2 shows that the pixel lens was designed by matching the subpixel size to the area of the viewing angle of the view point in the designed symmetrical FZP ring lens. We have considered two types of pixel lenses: circular and cylindrical^3^.

**Supplementary References**

1 Hecht, E. *Optics*, 5th edn. (Pearson, 2016).

2 Zheng, X. R *et al.* Highly efficient and ultra-broadband graphene oxide ultrathin lenses with three-dimensional subwavelength focusing. *Nature Communications* **6**, 8433, (2015).

3 Deng, S. *et al.* Graphene nanoribbon based plasmonic Fresnel zone plate lenses. *RSC Advances* **7**, 16594-16601, (2017).
